# *catena*-Poly[[trimethyl­tin(IV)]-μ-2-phenyl­butano­ato]

**DOI:** 10.1107/S1600536810047835

**Published:** 2010-11-24

**Authors:** Baoying Zhang, Rufen Zhang

**Affiliations:** a College of Chemistry and Chemical Engineering, Liaocheng University, Shandong 252059, People’s Republic of China

## Abstract

In the title polymeric coordination compound, [Sn(CH_3_)_3_(C_10_H_11_O_2_)]_*n*_, the Sn atom has a distorted trigonal–bipyramidal coordination geometry, with two O atoms of two symmetry-related carboxyl­ate ligands in axial positions and three methyl groups in equatorial positions. In the crystal structure, carboxyl­ate bridges link the metal atoms, forming zigzag chains parallel to the *b* axis.

## Related literature

For the biological activity of organotin compounds, see: Dubey & Roy (2003 ▶). For a related structure, see: Ma *et al.* (2008 ▶). 
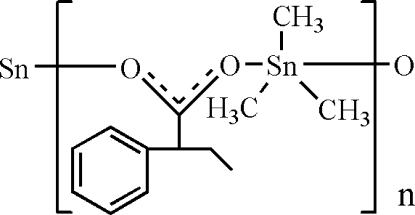


## Experimental

### 

#### Crystal data

[Sn(CH_3_)_3_(C_10_H_11_O_2_)]
*M* *_r_* = 326.98
Monoclinic, 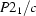

*a* = 11.0872 (12) Å
*b* = 10.0385 (9) Å
*c* = 13.2736 (15) Å
β = 103.828 (1)°
*V* = 1434.5 (3) Å^3^
*Z* = 4
Mo *K*α radiation
μ = 1.77 mm^−1^
*T* = 298 K
0.46 × 0.43 × 0.35 mm

#### Data collection

Siemens SMART CCD area-detector diffractometer
Absorption correction: multi-scan (*SADABS*; Sheldrick, 1996 ▶) *T*_min_ = 0.497, *T*_max_ = 0.577
6977 measured reflections
2512 independent reflections
2027 reflections with *I* > 2σ(*I*)
*R*_int_ = 0.027

#### Refinement

*R*[*F* ^2^ > 2σ(*F* ^2^)] = 0.023
*wR*(*F* ^2^) = 0.060
*S* = 1.14
2512 reflections
149 parameters
H-atom parameters constrained
Δρ_max_ = 0.88 e Å^−3^
Δρ_min_ = −0.50 e Å^−3^

### 

Data collection: *SMART* (Siemens, 1996 ▶); cell refinement: *SAINT* (Siemens, 1996 ▶); data reduction: *SAINT*; program(s) used to solve structure: *SHELXS97* (Sheldrick, 2008 ▶); program(s) used to refine structure: *SHELXL97* (Sheldrick, 2008 ▶); molecular graphics: *SHELXTL* (Sheldrick, 2008 ▶); software used to prepare material for publication: *SHELXTL*.

## Supplementary Material

Crystal structure: contains datablocks I, global. DOI: 10.1107/S1600536810047835/rz2525sup1.cif

Structure factors: contains datablocks I. DOI: 10.1107/S1600536810047835/rz2525Isup2.hkl

Additional supplementary materials:  crystallographic information; 3D view; checkCIF report

## Figures and Tables

**Fig. 1.**
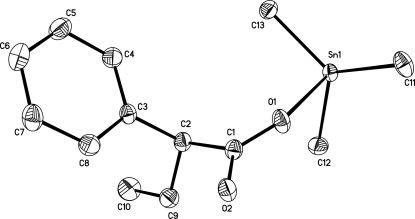
The asymmetric unit of the title compound, showing 50% probability displacement ellipsoids. H atoms are omitted for clarity.

**Fig. 2.**
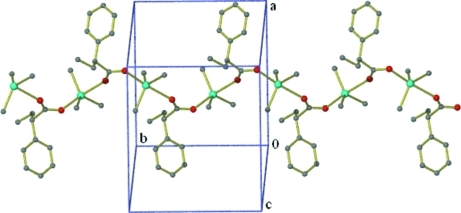
Partial packing diagram of the title compound showing a polymeric chain parallel to the b axis. Hydrohen atoms are omitted for clarity.

## supplementary crystallographic information

### Comment

In recent years, organotin compounds have been attracting more and more attention due to their wide range of industrial applications and biological activities (Dubey & Roy, 2003). As a part of our ongoing investigations in this field, we have synthesized the title compound and present its crystal structure here.

The asymmetric unit of the title compound is shown in Fig. 1. An extended one-dimensional zigzag chain structure running parallel to the *b* axis is formed by the bridging role of  the 2-phenyl-butyrate anions (Fig. 2). The Sn—O bond distances in the compound (Sn1—O1 = 2.446 (2) Å; Sn1—O2^i^ = 2.173 (2) Å; symmetry code: (i): 1-x, 1/2+y, 1/2-z) are comparable to those found in a related organotin carboxylate (Ma *et al.*, 2008). The Sn atom is five-coordinate in a slightly distorted trigonal-bipyramidal coordination geometry, provided by and the methyl groups in the equatorial positions and two O atoms of symmetry related carboxylate groups in the axial positions.

### Experimental

The reaction was carried out under a nitrogen atmosphere. 2-Phenyl-butyric acid (1 mmol) and sodium ethoxide (1 mmol) were added to a stirred solution of benzene (30 ml) in a Schlenk flask and stirred for 0.5 h. Trimethyltin chloride (1 mmol) was then added to the reactor and the reaction mixture was stirred for 12 h at room temperature. The resulting clear solution was evaporated under vacuum. The product was crystallized from a solution of diethyl ether to yield colourless block-like crystals of the title compound (yield 78%). Anal. Calcd (%) for C_13_H_20_O_2_Sn_1_ (Mr = 328.05): *C*,47.75; H, 6.16. Found (%): C, 47.56; H, 6.29.

### Refinement

The H atoms were positioned geometrically, with methyl C—H distances of 0.96Å and aromatic C—H distances of 0.93 Å, and refined as riding on their parent atoms, with *U*_iso_(H) = 1.2 *U*_eq_(C) or 1.5 *U*_eq_(C) for the methyl groups.

### Crystal data

**Table:**

[Sn(CH_3_)_3_(C_10_H_11_O_2_)]	*F*(000) = 656
*M**_r_* = 326.98	*D*_x_ = 1.514 Mg m^−^^3^
Monoclinic, *P*2_1_/*c*	Mo *K*α radiation, λ = 0.71073 Å
Hall symbol: -P 2ybc	Cell parameters from 3646 reflections
*a* = 11.0872 (12) Å	θ = 2.2–27.1°
*b* = 10.0385 (9) Å	µ = 1.77 mm^−^^1^
*c* = 13.2736 (15) Å	*T* = 298 K
β = 103.828 (1)°	Block, colourless
*V* = 1434.5 (3) Å^3^	0.46 × 0.43 × 0.35 mm
*Z* = 4	

### Data collection

**Table:**

Siemens SMART CCD area-detector diffractometer	2512 independent reflections
Radiation source: fine-focus sealed tube	2027 reflections with *I* > 2σ(*I*)
graphite	*R*_int_ = 0.027
phi and ω scans	θ_max_ = 25.0°, θ_min_ = 1.9°
Absorption correction: multi-scan (*SADABS*; Sheldrick, 1996)	*h* = −13→10
*T*_min_ = 0.497, *T*_max_ = 0.577	*k* = −11→11
6977 measured reflections	*l* = −15→15

### Refinement

**Table:**

Refinement on *F*^2^	Primary atom site location: structure-invariant direct methods
Least-squares matrix: full	Secondary atom site location: difference Fourier map
*R*[*F*^2^ > 2σ(*F*^2^)] = 0.023	Hydrogen site location: inferred from neighbouring sites
*wR*(*F*^2^) = 0.060	H-atom parameters constrained
*S* = 1.14	*w* = 1/[σ^2^(*F*_o_^2^) + (0.0194*P*)^2^ + 0.8554*P*] where *P* = (*F*_o_^2^ + 2*F*_c_^2^)/3
2512 reflections	(Δ/σ)_max_ < 0.001
149 parameters	Δρ_max_ = 0.88 e Å^−^^3^
0 restraints	Δρ_min_ = −0.50 e Å^−^^3^

### Special details

**Table:**

Geometry. All e.s.d.'s (except the e.s.d. in the dihedral angle between two l.s. planes) are estimated using the full covariance matrix. The cell e.s.d.'s are taken into account individually in the estimation of e.s.d.'s in distances, angles and torsion angles; correlations between e.s.d.'s in cell parameters are only used when they are defined by crystal symmetry. An approximate (isotropic) treatment of cell e.s.d.'s is used for estimating e.s.d.'s involving l.s. planes.
Refinement. Refinement of *F*^2^ against ALL reflections. The weighted *R*-factor *wR* and goodness of fit *S* are based on *F*^2^, conventional *R*-factors *R* are based on *F*, with *F* set to zero for negative *F*^2^. The threshold expression of *F*^2^ > σ(*F*^2^) is used only for calculating *R*-factors(gt) *etc*. and is not relevant to the choice of reflections for refinement. *R*-factors based on *F*^2^ are statistically about twice as large as those based on *F*, and *R*- factors based on ALL data will be even larger.

### Fractional atomic coordinates and isotropic or equivalent isotropic displacement parameters (Å^2^)

**Table:**

	*x*	*y*	*z*	*U*_iso_*/*U*_eq_	
Sn1	0.44402 (2)	0.40204 (2)	0.201458 (18)	0.03753 (9)	
O1	0.5747 (2)	0.2100 (2)	0.2682 (2)	0.0540 (7)	
O2	0.6875 (2)	0.0565 (2)	0.3670 (2)	0.0491 (6)	
C1	0.6678 (3)	0.1777 (3)	0.3364 (3)	0.0416 (8)	
C2	0.7630 (3)	0.2802 (3)	0.3881 (3)	0.0429 (8)	
H2	0.7332	0.3676	0.3597	0.052*	
C3	0.8877 (3)	0.2555 (3)	0.3620 (3)	0.0398 (8)	
C4	0.9310 (4)	0.3421 (4)	0.2984 (3)	0.0532 (10)	
H4	0.8832	0.4157	0.2712	0.064*	
C5	1.0441 (4)	0.3220 (5)	0.2740 (3)	0.0644 (12)	
H5	1.0713	0.3812	0.2302	0.077*	
C6	1.1157 (4)	0.2158 (4)	0.3139 (4)	0.0625 (11)	
H6	1.1917	0.2023	0.2974	0.075*	
C7	1.0759 (4)	0.1288 (4)	0.3784 (4)	0.0605 (11)	
H7	1.1252	0.0568	0.4067	0.073*	
C8	0.9617 (4)	0.1485 (4)	0.4013 (3)	0.0539 (10)	
H8	0.9344	0.0882	0.4442	0.065*	
C9	0.7729 (3)	0.2846 (4)	0.5052 (3)	0.0542 (10)	
H9A	0.6904	0.2941	0.5171	0.065*	
H9B	0.8071	0.2009	0.5358	0.065*	
C10	0.8547 (4)	0.3992 (4)	0.5590 (4)	0.0714 (13)	
H10A	0.9387	0.3852	0.5540	0.107*	
H10B	0.8521	0.4025	0.6307	0.107*	
H10C	0.8245	0.4818	0.5260	0.107*	
C11	0.3315 (4)	0.2560 (4)	0.1071 (3)	0.0580 (11)	
H11A	0.3437	0.1713	0.1416	0.087*	
H11B	0.3542	0.2497	0.0419	0.087*	
H11C	0.2457	0.2813	0.0952	0.087*	
C12	0.4289 (4)	0.4269 (4)	0.3558 (3)	0.0585 (11)	
H12A	0.5019	0.4708	0.3954	0.088*	
H12B	0.4208	0.3413	0.3857	0.088*	
H12C	0.3570	0.4800	0.3564	0.088*	
C13	0.5938 (3)	0.4815 (4)	0.1461 (3)	0.0537 (10)	
H13A	0.5620	0.5422	0.0902	0.081*	
H13B	0.6373	0.4104	0.1218	0.081*	
H13C	0.6496	0.5279	0.2014	0.081*	

### Atomic displacement parameters (Å^2^)

**Table:**

	*U*^11^	*U*^22^	*U*^33^	*U*^12^	*U*^13^	*U*^23^
Sn1	0.03457 (14)	0.03491 (14)	0.04067 (16)	0.00008 (11)	0.00416 (10)	−0.00243 (11)
O1	0.0432 (15)	0.0420 (14)	0.0667 (18)	0.0031 (12)	−0.0065 (13)	0.0054 (13)
O2	0.0419 (14)	0.0321 (13)	0.0641 (18)	−0.0046 (10)	−0.0052 (12)	0.0063 (12)
C1	0.037 (2)	0.038 (2)	0.049 (2)	0.0002 (15)	0.0094 (17)	−0.0024 (17)
C2	0.0395 (19)	0.0355 (19)	0.052 (2)	−0.0022 (15)	0.0075 (17)	0.0012 (17)
C3	0.0360 (18)	0.0355 (19)	0.044 (2)	−0.0047 (15)	0.0020 (16)	−0.0059 (16)
C4	0.050 (2)	0.049 (2)	0.060 (3)	−0.0004 (18)	0.012 (2)	0.008 (2)
C5	0.059 (3)	0.067 (3)	0.073 (3)	−0.006 (2)	0.027 (2)	0.008 (2)
C6	0.045 (2)	0.063 (3)	0.081 (3)	−0.004 (2)	0.019 (2)	−0.013 (2)
C7	0.049 (2)	0.051 (2)	0.080 (3)	0.0110 (19)	0.012 (2)	0.001 (2)
C8	0.053 (2)	0.047 (2)	0.062 (3)	0.0039 (18)	0.014 (2)	0.010 (2)
C9	0.053 (2)	0.056 (2)	0.054 (2)	0.001 (2)	0.0138 (19)	0.000 (2)
C10	0.083 (3)	0.067 (3)	0.063 (3)	−0.010 (2)	0.015 (2)	−0.022 (2)
C11	0.061 (2)	0.042 (2)	0.059 (3)	0.0054 (19)	−0.010 (2)	−0.0134 (19)
C12	0.064 (3)	0.066 (3)	0.048 (2)	−0.002 (2)	0.018 (2)	−0.002 (2)
C13	0.044 (2)	0.060 (2)	0.059 (3)	0.0031 (19)	0.017 (2)	0.010 (2)

### Geometric parameters (Å, °)

**Table:**

Sn1—C12	2.110 (4)	C6—H6	0.9300
Sn1—C13	2.125 (3)	C7—C8	1.385 (5)
Sn1—C11	2.128 (3)	C7—H7	0.9300
Sn1—O2^i^	2.173 (2)	C8—H8	0.9300
Sn1—O1	2.446 (2)	C9—C10	1.532 (5)
O1—C1	1.243 (4)	C9—H9A	0.9700
O2—C1	1.285 (4)	C9—H9B	0.9700
O2—Sn1^ii^	2.173 (2)	C10—H10A	0.9600
C1—C2	1.516 (5)	C10—H10B	0.9600
C2—C3	1.524 (5)	C10—H10C	0.9600
C2—C9	1.533 (5)	C11—H11A	0.9600
C2—H2	0.9800	C11—H11B	0.9600
C3—C4	1.376 (5)	C11—H11C	0.9600
C3—C8	1.376 (5)	C12—H12A	0.9600
C4—C5	1.384 (5)	C12—H12B	0.9600
C4—H4	0.9300	C12—H12C	0.9600
C5—C6	1.359 (6)	C13—H13A	0.9600
C5—H5	0.9300	C13—H13B	0.9600
C6—C7	1.368 (6)	C13—H13C	0.9600
			
C12—Sn1—C13	122.76 (17)	C8—C7—H7	120.2
C12—Sn1—C11	118.78 (16)	C3—C8—C7	121.4 (4)
C13—Sn1—C11	116.88 (17)	C3—C8—H8	119.3
C12—Sn1—O2^i^	96.92 (13)	C7—C8—H8	119.3
C13—Sn1—O2^i^	95.12 (12)	C10—C9—C2	112.7 (3)
C11—Sn1—O2^i^	90.26 (12)	C10—C9—H9A	109.1
C12—Sn1—O1	85.38 (13)	C2—C9—H9A	109.1
C13—Sn1—O1	88.69 (12)	C10—C9—H9B	109.1
C11—Sn1—O1	83.32 (12)	C2—C9—H9B	109.1
O2^i^—Sn1—O1	173.50 (8)	H9A—C9—H9B	107.8
C1—O1—Sn1	141.9 (2)	C9—C10—H10A	109.5
C1—O2—Sn1^ii^	119.8 (2)	C9—C10—H10B	109.5
O1—C1—O2	121.8 (3)	H10A—C10—H10B	109.5
O1—C1—C2	121.3 (3)	C9—C10—H10C	109.5
O2—C1—C2	116.9 (3)	H10A—C10—H10C	109.5
C1—C2—C3	111.0 (3)	H10B—C10—H10C	109.5
C1—C2—C9	110.6 (3)	Sn1—C11—H11A	109.5
C3—C2—C9	112.7 (3)	Sn1—C11—H11B	109.5
C1—C2—H2	107.4	H11A—C11—H11B	109.5
C3—C2—H2	107.4	Sn1—C11—H11C	109.5
C9—C2—H2	107.4	H11A—C11—H11C	109.5
C4—C3—C8	117.5 (3)	H11B—C11—H11C	109.5
C4—C3—C2	120.3 (3)	Sn1—C12—H12A	109.5
C8—C3—C2	122.1 (3)	Sn1—C12—H12B	109.5
C3—C4—C5	121.4 (4)	H12A—C12—H12B	109.5
C3—C4—H4	119.3	Sn1—C12—H12C	109.5
C5—C4—H4	119.3	H12A—C12—H12C	109.5
C6—C5—C4	120.0 (4)	H12B—C12—H12C	109.5
C6—C5—H5	120.0	Sn1—C13—H13A	109.5
C4—C5—H5	120.0	Sn1—C13—H13B	109.5
C5—C6—C7	120.0 (4)	H13A—C13—H13B	109.5
C5—C6—H6	120.0	Sn1—C13—H13C	109.5
C7—C6—H6	120.0	H13A—C13—H13C	109.5
C6—C7—C8	119.7 (4)	H13B—C13—H13C	109.5
C6—C7—H7	120.2		

Symmetry codes: (i) −*x*+1, *y*+1/2, −*z*+1/2; (ii) −*x*+1, *y*−1/2, −*z*+1/2.

## References

[bb1] Dubey, S. K. & Roy, U. (2003). *Appl. Organomet. Chem.* **17**, 3–8.

[bb2] Ma, C., Wang, Q. & Zhang, R. (2008). *Eur. J. Inorg. Chem.* pp. 1926–1934.

[bb3] Sheldrick, G. M. (1996). *SADABS* University of Göttingen, Germany.

[bb4] Sheldrick, G. M. (2008). *Acta Cryst.* A**64**, 112–122. 10.1107/S0108767307043930 18156677

[bb5] Siemens (1996). *SMART* and *SAINT* Siemens Analytical X-ray Instruments Inc., Madison, Wisconsin, USA.

